# Raman and Infrared Signatures of Layered Boron Nitride Polytypes: A First-Principles Study

**DOI:** 10.3390/nano15201567

**Published:** 2025-10-15

**Authors:** Priyanka Mishra, Nevill Gonzalez Szwacki

**Affiliations:** Faculty of Physics, University of Warsaw, Pasteura 5, PL-02093 Warsaw, Poland; p.mishra3@student.uw.edu.pl

**Keywords:** boron nitride, polytypes, first-principles calculations, vibrational properties, Raman/infrared spectroscopy

## Abstract

We present a study based on first-principles calculations of the vibrational and spectroscopic properties of four types of layered boron nitride (BN) polymorphs: *e*-BN (AA), *h*-BN (${\mathrm{AA}}^{\prime }$), *r*-BN (ABC), and *b*-BN (AB). By using density functional perturbation theory with van der Waals corrections, we calculate phonon frequencies and Raman/infrared (IR) activities at the Γ point and extract specific spectral fingerprints for each stack. In *e*-BN, we observe a sharp, isolated high-frequency $E^{\prime }$ mode at $1420.9{\mathrm{cm}}^{-1}$ that is active in both Raman and IR. For *h*-BN, the characteristic Raman $E_{2g}$ line occurs at $1415.5{\mathrm{cm}}^{-1}$. The out-of-plane IR-active $A_{2u}$ branch shows a mid-frequency TO/LO pair at 673.5/$806.6{\mathrm{cm}}^{-1}$, which closely matches experimental results. Rhombohedral *r*-BN has a strong, coincident Raman/IR high-frequency feature (*E*) at $1418.5{\mathrm{cm}}^{-1}$, along with a large IR LO partner at $1647.3{\mathrm{cm}}^{-1}$, consistent with observed Raman and IR signatures. Bernal *b*-BN displays the most complicated pattern. It combines a robust mid-frequency $A_{2}^{\prime \prime }$ pair (TO/LO at 697.9/$803.5{\mathrm{cm}}^{-1}$) with multiple high-frequency $E^{\prime }$ modes (TO near 1416.9 and $1428.1{\mathrm{cm}}^{-1}$, each with LO counterparts). These stack-dependent Raman and IR fingerprints match existing experimental data for *h*-BN and *r*-BN and provide clear predictions for *e*-BN and *b*-BN. The results offer a consistent framework for identifying and interpreting vibrational spectra in layered $sp^{2}$ boron nitride and related materials.

## 1. Introduction

Hexagonal boron nitride (*h*-BN) is a prototypical layered material with a honeycomb lattice structure analogous to graphite, composed of strongly bonded in-plane B–N atoms ($sp^{2}$ hybridization) and weakly interacting interlayer planes. This structural anisotropy imparts *h*-BN with a wide band gap, exceptional thermal and chemical stability, and a rich vibrational spectrum, positioning it as a vital material in diverse applications including deep ultraviolet optoelectronics, two-dimensional (2D) heterostructures, and quantum light emission [1,2]. One of the most intriguing and technologically relevant aspects of *h*-BN is its tendency to form various stacking sequences, or polytypes, due to the nearly degenerate interlayer energy landscape. These polytypes—notably ${\mathrm{AA}}^{\prime }$ (*h*-BN), ABC (*r*-BN), AA (*e*-BN), and AB (*b*-BN)—differ only in how their layers are arranged along the out-of-plane direction, yet exhibit markedly distinct electronic, vibrational, and optical properties [2,3,4,5,6]. The co-existence of these polytypes within single crystals leads to structural disorder, interfaces, and stacking faults, which can affect charge transport, phonon lifetimes, and emission characteristics [3,7].

Despite extensive research, the thermodynamic and vibrational stability of boron nitride (BN) polymorphs remains a topic of active investigation and debate. While cubic BN (*c*-BN) is the ground state under high pressure, recent high-level calculations and experiments suggest that *h*-BN is thermodynamically stabilized at ambient conditions through entropic contributions and many-body van der Waals (vdW) interactions [8,9]. In particular, the subtle energy differences between the low-energy $sp^{2}$ phases (such as *h*-BN and *r*-BN) are on the order of tens of meV per formula unit–a scale that demands theoretical methods beyond conventional density functional theory (DFT) to resolve [8,9,10]. Including nonlocal correlation effects is thus essential for capturing the relative stability and interlayer coupling in these materials. Stacking order is not merely a structural detail but a critical determinant of the electronic structure and vibrational response. For instance, ${\mathrm{AA}}^{\prime }$ stacking (common in *h*-BN) is predicted to be the lowest energy configuration among the layered forms [11]. In contrast, ABC stacking (*r*-BN) yields distinct optical and electronic properties, including a redshifted conduction band minimum and richer infrared (IR) and Raman activity [3,4,6]. Additionally, *b*-BN and *e*-BN represent higher-symmetry and more idealized stackings (AB and AA, respectively), which, while less studied experimentally, serve as critical structural models to explore the influence of interlayer registry and symmetry on physical properties [5].

Experimental studies using cathodoluminescence, Raman scattering, X-ray absorption, UV photoluminescence, and IR spectroscopy have revealed that polytypism dramatically influences the optoelectronic response of BN [2,3,7,12]. Notably, recent photoluminescence (PL) studies have succeeded in distinguishing BN polytypes through subtle spectral shifts and emission signatures [12,13]. However, interpreting these data is complicated by the frequent co-existence of polytypes, nanoscale disorder, and temperature-induced transformations [8,9]. In this context, vibrational spectroscopy, particularly Raman and IR techniques, has emerged as a powerful diagnostic tool for identifying BN polytypes, owing to its sensitivity to symmetry, stacking order, and interlayer coupling.

To support this approach, computational modeling plays a pivotal role in interpreting vibrational spectra and guiding polytype-specific material characterization. In this work, we present a comprehensive first-principles investigation of the vibrational and dielectric properties of four BN polymorphs: *e*-BN (AA), *h*-BN (${\mathrm{AA}}^{\prime }$), *r*-BN (ABC), and *b*-BN (AB). We use density functional perturbation theory (DFPT) with and without vdW corrections to examine the stacking-dependent evolution of phonon frequencies, Raman and infrared activities, and dielectric properties. Our results provide detailed spectroscopic fingerprints-including irreducible representations and intensities-that enable clear identification of BN polytypes. Understanding these intricate relationships is crucial for the fundamental science of 2D materials and for engineering BN-based optoelectronic and quantum devices with targeted performance. The findings presented here aim to bridge this gap and contribute to a more predictive understanding of polytype-dependent vibrational behavior in layered BN.

## 2. Computational Details

**Figure 1:**
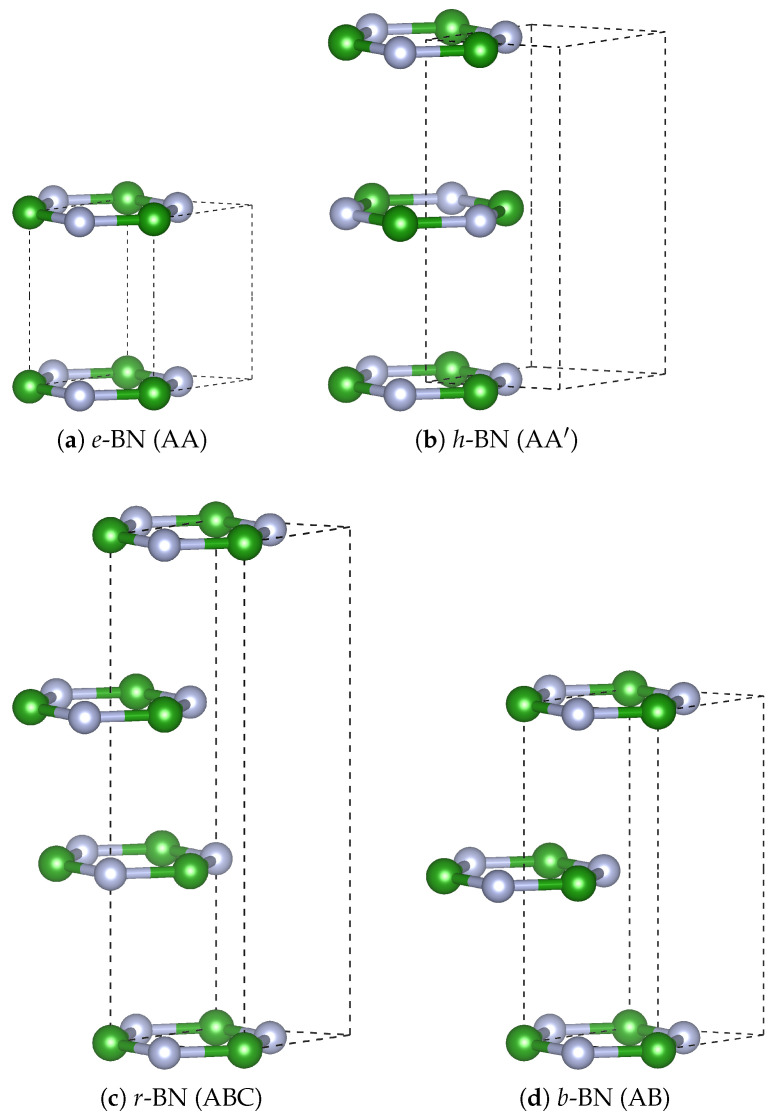
Crystal structures of BN polymorphs (green = B, gray = N): (**a**) *e*-BN [$P\bar{6}m2$ (187)], (**b**) *h*-BN [$P6_{3}/mmc$ (194)], (**c**) *r*-BN [R3m (160)], and (**d**) *b*-BN [$P\bar{6}m2$ (187)].

We employed DFT, as implemented in the Quantum ESPRESSO (QE 7.2) package, to investigate the structural, electronic, and vibrational properties of different BN phases. The calculations were initiated using the experimental unit cell, with explicitly defined lattice parameters in Cartesian coordinates. A plane-wave energy cutoff of 80 Ry and a charge-density cutoff of 320 Ry were used to ensure numerical accuracy, following a systematic convergence analysis. The exchange–correlation interactions were treated using the Perdew–Burke–Ernzerhof (PBE) functional within the generalized gradient approximation (GGA). Brillouin zone sampling was performed using a Γ-centered 16×16×16 Monkhorst–Pack *k*-point mesh to ensure accurate integration. Given the layered nature of BN, vdW corrections were incorporated using the Grimme-D2 (DFT-D2) scheme as implemented in QE [14]. The convergence criteria were set to $1.0\times 10^{-5}$ Ry for total energy and $1.0\times 10^{-4}$ Ry/Bohr for atomic forces. Projector-augmented wave (PAW) pseudopotentials (taken from the PSLibrary included in QE) were employed for electron–ion interactions, providing a reliable description of core-valence interactions while maintaining computational efficiency. All electronic structure calculations used fixed (insulating) occupations (no smearing). The cohesive energy per atom, $E_{c}$, was calculated as $E_{c}=\frac{n_{B}E_{B}^{\mathrm{atom}\;}+n_{N}E_{N}^{\mathrm{atom}\;}-E_{\mathrm{BN}}}{N},$ where $E_{B}^{\mathrm{atom}\;}$ and $E_{N}^{\mathrm{atom}\;}$ are the total energies of isolated boron and nitrogen atoms, respectively; $n_{B}$ and $n_{N}$ are the numbers of B and N atoms in the BN unit cell; $E_{\mathrm{BN}}$ is the total energy of the BN polymorph; and $N=n_{B}+n_{N}$ is the total number of atoms. The atomic energies were computed in large cubic cells to eliminate spurious interactions. Structural and band properties were computed with PBE/PAW + D2, while vibrational Raman/IR intensities were obtained using PW/ONCV + D2 (for details see Section 3.4). For structural and electronic properties of *r*-BN, we used the unit cell shown in Figure 1c, whereas vibrational properties were computed using the primitive rhombohedral cell.

## 3. Results

### 3.1. Crystal Structures for Different Polymorphs of BN

The atomic structures of four layered BN polymorphs–*e*-BN (AA), *h*-BN (${\mathrm{AA}}^{\prime }$), *r*-BN (ABC), and *b*-BN (AB) are illustrated in Figure 1a–d. These polymorphs differ in stacking sequences, symmetry groups, and interlayer arrangements. Structural parameters, cohesive energies ($E_{c}$), and electronic band gaps (*E*) are summarized in Table 1. The stacking order is a key factor controlling the interlayer spacing and stability of these $sp^{2}$-bonded systems.

*e*-BN (AA) crystallizes in the non-centrosymmetric space group $P\bar{6}m2$ (No. 187) and features direct AA stacking, where boron and nitrogen atoms are aligned vertically across layers (Figure 1a). This configuration leads to weak interlayer bonding in the absence of vdW corrections. Inclusion of vdW interactions significantly contracts the *c*-axis from 5.06 to 3.38 Å, as shown in Table 1, improving agreement with values reported by Gil et al. [2]. No experimental data exist for this stacking, but theoretical values are consistent across methods.

*h*-BN (${\mathrm{AA}}^{\prime }$) is the most commonly observed polymorph, adopting the $P6_{3}/mmc$ (No. 194) space group symmetry, with alternating B and N atoms stacked in a staggered bilayer configuration (Figure 1b). The calculated lattice constants with vdW corrections (a=2.512 Å, c=6.179 Å) agree well with both theoretical and experimental values reported in Table 1 of Gil et al. [2] and Ahmed et al. [15], where a=2.478 Å and c=6.354 Å are cited as experimental averages. This stacking is energetically favored and widely accepted as the most stable $sp^{2}$-BN structure under ambient conditions.

*r*-BN (ABC) belongs to the rhombohedral space group R3m (No. 160) and exhibits a three-layer ABC stacking sequence (Figure 1c). The calculated interlayer distance (c=9.17 Å with vdW) aligns reasonably with experimental reports (c=9.679 Å) in Table 1 of Gil et al. [2]. Our calculations also show a slightly larger cohesive energy compared to *h*-BN (7.207 vs. 7.205 eV), consistent with the finding by Nikaido et al. [9] that *r*-BN and *h*-BN are nearly degenerate in energy, although *h*-BN remains thermodynamically most stable at 0 K.

*b*-BN (AB) is a less commonly studied polymorph with $P\bar{6}m2$ (No. 187) symmetry, characterized by a two-layer AB stacking, also called Bernal stacking (Figure 1d). The optimized lattice parameters with vdW corrections (a=2.511 Å, c=6.117 Å) fall within the range of those reported for $sp^{2}$-BN systems in Gil et al. [2]. The relative stability of *b*-BN is comparable to *r*-BN in our calculations, and it exhibits a direct band gap, suggesting unique electronic properties discussed in Section 3.2.

Across all polymorphs, we observe a strong sensitivity of the *c*-axis lattice parameter to vdW corrections, while the in-plane lattice constant *a* remains nearly invariant. This agrees with trends reported in both Gil et al. [2] and Ahmed et al. [15], whose comprehensive tables include both theoretical and experimental lattice data. The cohesive energies calculated here suggest a delicate balance in stability among $sp^{2}$ BN polytypes, with *h*-BN slightly preferred, in line with diffusion Monte Carlo results by Nikaido et al. [9]. These results highlight the essential role of interlayer stacking in modulating structural and energetic properties of layered BN and provide a consistent theoretical framework in support of experimental data. Overall, the structural polymorphism in $sp^{2}$-bonded BN results in a delicate balance between symmetry, interlayer stacking, and vdW interactions, all of which are crucial for understanding the phase stability and physical properties of BN-based materials.

### 3.2. Electronic Band Structure

**Figure 2:**
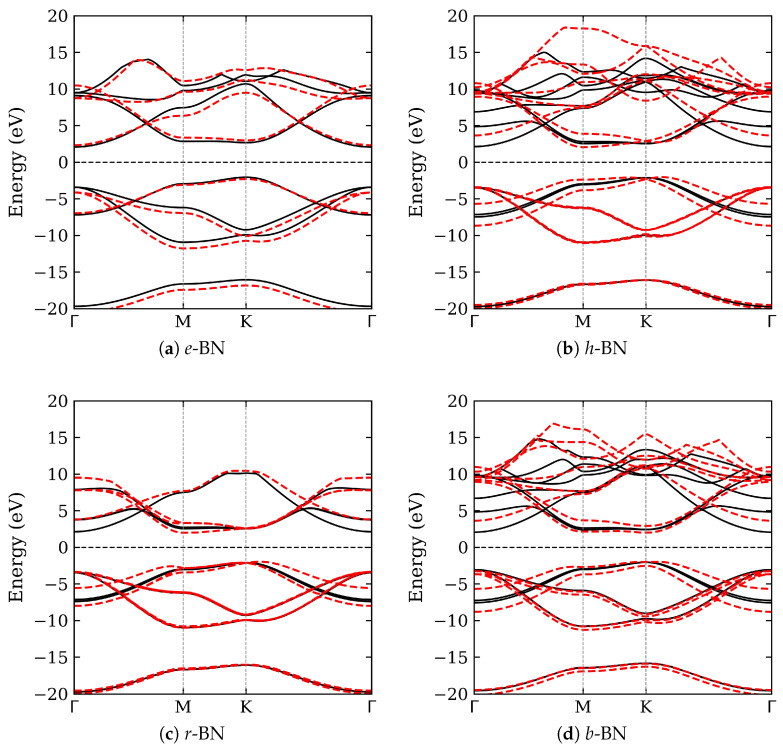
Electronic band structures of the studied BN polymorphs with and without vdW corrections. Black solid lines indicate results without vdW, and red dashed lines include vdW corrections. Panels: (**a**) *e*-BN (AA), (**b**) *h*-BN (${\mathrm{AA}}^{\prime }$), (**c**) *r*-BN (ABC), and (**d**) *b*-BN (AB).

The electronic band structures of the four studied BN polymorphs were calculated with and without the vdW correction. The results are shown in Figure 2, while a summary of the band gaps’ details is presented in Table 1. All polymorphs exhibit wide band gaps in the range of 3.94 to 4.63 eV, consistent with the semiconducting nature of $sp^{2}$-bonded BN. The inclusion of vdW interactions significantly affects the out-of-plane lattice constants and, consequently, the electronic band structure, underscoring the importance of capturing weak interlayer forces in layered materials. Across the studied BN polymorphs, the electronic band structure reveals predominantly indirect band gaps, except for *b*-BN, which exhibits a direct transition at the K point. Specifically, *e*-BN, *h*-BN, and *r*-BN show indirect band gaps ranging from 3.94 to 4.63 eV, typically between the K and Γ or K and M points. Among them, *e*-BN has the widest gap (4.63 eV), while *r*-BN has the narrowest (3.94 eV), highlighting the influence of stacking geometry and interlayer coupling. The reduced gap in *r*-BN, a rhombohedral phase, can be attributed to enhanced interlayer orbital overlap due to its three-layer ABC stacking, which modifies the conduction band minimum. In contrast, *b*-BN exhibits a direct band gap (K–K), a property desirable for optoelectronic applications relying on vertical transitions. This distinction underscores the sensitivity of the electronic structure to interlayer interactions and symmetry, and emphasizes the potential of stacking-engineered BN for tailored band gap applications.

Comparison with earlier work by Ahmed et al. [15] shows qualitative agreement in the trends of band gap variations across BN polymorphs. Their full-potential LAPW calculations using the Engel-Vosko GGA functional yielded a gap of 4.18 eV for *h*-BN and 4.21 eV for *r*-BN, closely matching our PBE/PAW + D2 results. However, Ahmed et al. emphasize the importance of using GGA-EV to better align with experimental values, suggesting a pathway for further refinement. Additional insight is provided by Olovsson and Magnuson [3], who investigated *h*-, *r*-, and turbostratic BN using X-ray absorption near-edge structure (XANES) spectroscopy. Their DFT+core-hole simulations revealed distinctive $\pi^{*}$ and $\sigma^{*}$ features sensitive to stacking order. The observed shift in the $\pi^{*}$ onset in *r*-BN relative to *h*-BN correlates with our finding of a narrower band gap in the rhombohedral structure. Moreover, their turbostratic BN models exhibited an average band gap of approximately 3.86 eV, further supporting the notion that stacking disorder can be exploited to engineer BN’s electronic properties.

Lastly, our results show that interlayer stacking and vdW interactions are critical determinants of the electronic properties of BN polymorphs. The transition between indirect and direct band gaps, combined with variations in gap magnitude, offers opportunities for targeted design of BN-based materials in optoelectronics, UV photonics, and quantum applications.

### 3.3. Phonon Frequencies

The vibrational properties of layered BN polymorphs are susceptible to weak interlayer forces and stacking configurations. To accurately capture these effects, we computed phonon frequencies at the Γ point of the Brillouin zone using DFPT with and without vdW corrections, as implemented in QE. The refined phonon spectra–including full irreducible representation labeling and explicit identification of silent (S), infrared (I), and Raman (R) active modes are summarized in Table 2. All optical phonons are included, providing a comprehensive mode-by-mode comparison across BN polymorphs. Including vdW interactions significantly impacts phonon frequencies, particularly in polymorphs with strong interlayer coupling such as *h*-BN and *r*-BN. Consistent with prior studies [2,9], vdW corrections soften out-of-plane optical modes and bring theoretical spectra closer to experimental IR and Raman observations. The detailed mode analysis reveals prominent shifts upon vdW inclusion, especially for low-frequency modes below $200{\mathrm{cm}}^{-1}$ and high-frequency optical branches near $1350{\mathrm{cm}}^{-1}$. For example, in *h*-BN, the out-of-plane infrared-active $A_{2u}$ mode shifts from $781.1{\mathrm{cm}}^{-1}$ (no vdW) to $723{\mathrm{cm}}^{-1}$ (with vdW), while the silent $B_{1g}$ mode also softens notably. Similarly, *r*-BN exhibits rich vibrational behavior with multiple modes showing dual IR and Raman activity (labeled as I + R), especially between 730 and $800{\mathrm{cm}}^{-1}$, where vdW corrections shift the $A_{1}$ modes by 20–$40{\mathrm{cm}}^{-1}$. In *e*-BN, the phonon spectrum reflects the high symmetry and absence of staggered stacking. We observe fewer distinct branches and minimal splitting between optical modes. The dominant IR-active $A_{2}^{\prime \prime }$ and $E^{\prime }$ modes remain near $780{\mathrm{cm}}^{-1}$ and $1343{\mathrm{cm}}^{-1}$, respectively, with vdW corrections introducing only moderate shifts. Notably, silent modes, such as those transforming as $B_{1g}$ in analogous systems, are absent here due to symmetry constraints. Conversely, *b*-BN shows a broader vibrational landscape. Low-frequency IR-active modes shift from $55{\mathrm{cm}}^{-1}$ (no vdW) to $180{\mathrm{cm}}^{-1}$ (with vdW), reflecting stronger interlayer coupling. Intermediate-frequency $A_{1}$ and $E^{\prime }$ modes also emerge around 730–$800{\mathrm{cm}}^{-1}$, with rich IR and Raman activity reflecting the lower symmetry of the AB stack. The high-frequency Raman-active $E^{\prime }$ modes near $1350{\mathrm{cm}}^{-1}$ remain prominent and shift modestly upon vdW inclusion.

**Figure 3:**
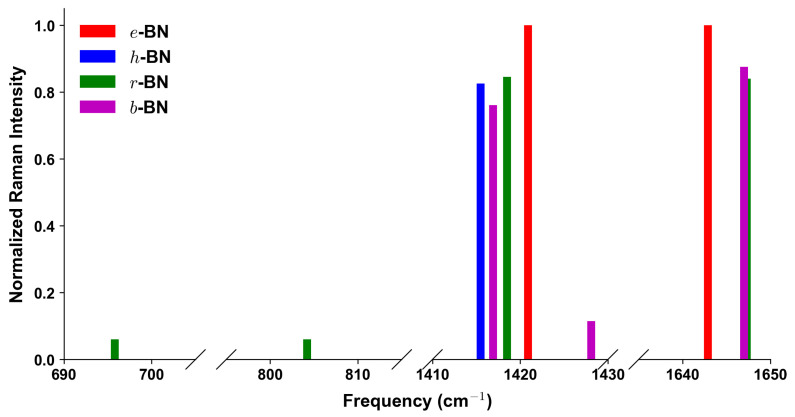
Computed Raman intensity spectra at the Γ point for the four BN polymorphs, obtained from PW/ONCV + D2 calculations. Intensities are normalized to the globally strongest peak among all polymorphs.

**Figure 4:**
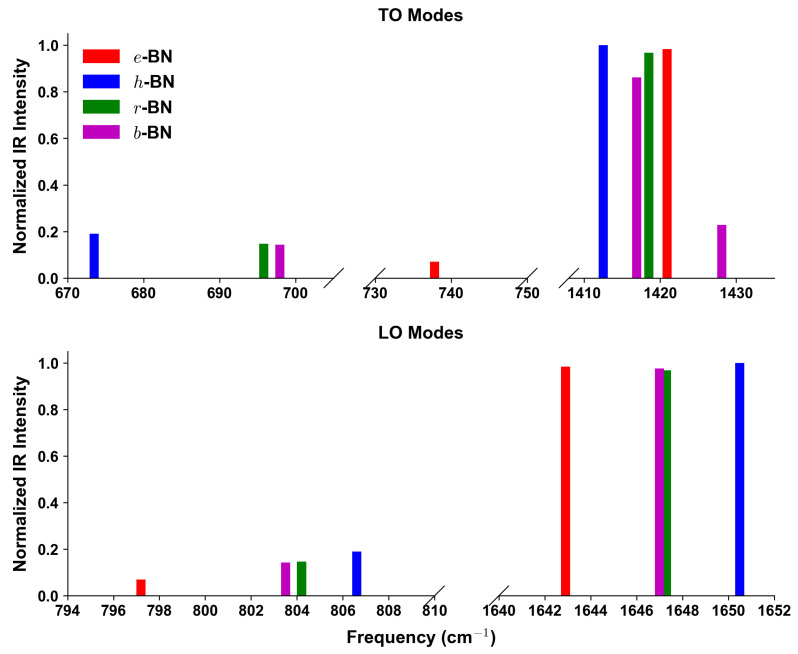
Computed infrared intensity spectra at the Γ point for the four BN polymorphs, obtained from PW/ONCV + D2 calculations. Intensities are normalized to the globally strongest peak across all polymorphs. The figure has two panels: top shows TO modes, bottom shows LO modes.

These results directly connect to the subsequent analysis of Raman and IR intensities (Figure 3 and Figure 4), where stacking-dependent features, such as the rich mid-frequency IR activity of *r*-BN and the sharper Raman peaks of *e*-BN, mirror the phonon characteristics detailed here. Overall, this detailed vibrational analysis highlights the critical role of vdW interactions and stacking order in shaping the phonon spectra of layered BN polymorphs. The combination of full irreducible representation labeling and activity classification provides a robust framework for both theoretical interpretation and experimental verification of BN polytypes.

### 3.4. Raman and Infrared Intensities

To obtain accurate IR and Raman intensities, pseudopotentials compatible with the linear and nonlinear response formalism in QE are essential. In particular, norm-conserving (NC) pseudopotentials within the local density approximation (LDA) of Perdew–Wang (PW) enable reliable Raman intensity calculations, as they provide the second-order response properties, such as polarizability derivatives, required for this task. We reoptimized the lattice parameters and atomic positions of each BN polymorph using optimized NC Vanderbilt (ONCV) pseudopotentials from the PseudoDojo project [16]. All calculations were performed in LDA, following the same cutoffs and convergence thresholds as described in Section 2. The resulting PW/ONCV + D2 phonon frequencies (see Table 3) follow the same mode ordering and show close quantitative agreement, within expected functional- and pseudopotential-dependent shifts, with the PBE/PAW + D2 results in Table 2. This comparison highlights both the robustness of the trends across computational setups and the importance of using consistent pseudopotentials and exchange–correlation functionals when modeling spectroscopic properties of layered materials.

Since Raman and IR activities from DFPT in QE are reported in arbitrary units, all four BN polymorphs were computed with identical pseudopotentials, cutoffs, and convergence criteria, ensuring consistent scaling. For direct comparison, spectra were normalized to the globally strongest peak among all polymorphs, so that relative intensities across different stackings are represented on the same scale. The normalization was carried out per formula unit, allowing intensities to be compared consistently across different polymorphs. Figure 3 and Figure 4 display the Raman and IR intensity spectra at the Γ point. In the panels, only peaks with intensities larger than 0.01 are shown. Complete listings are provided in Table 4.

From Figure 3 and Figure 4, and Table 4, distinct fingerprints of the four BN polytypes can be identified. For *e*-BN, the defining feature is a sharp and isolated high-frequency $E^{\prime }$ mode at $1420.9{\mathrm{cm}}^{-1}$, which is both Raman- and IR-active (R ≈ 1.00; IR ≈ 0.98). The IR spectra show a pronounced LO–TO splitting of this mode (1420.9 vs. $1642.9{\mathrm{cm}}^{-1}$), exceeding $220{\mathrm{cm}}^{-1}$, while only weak IR-active features are present at mid frequencies (737.8 and $797.2{\mathrm{cm}}^{-1}$). In *h*-BN, the characteristic Raman signature is the $E_{2g}$ mode at $1415.5{\mathrm{cm}}^{-1}$ (R ≈ 0.83), and the IR response is dominated by the high-frequency $E_{1u}$ pair with TO/LO at 1412.5/$1650.5{\mathrm{cm}}^{-1}$ (both normalized IR ≈ 1.00). In addition, a unique mid-frequency $A_{2u}$ mode at $673.5{\mathrm{cm}}^{-1}$ (IR ≈ 0.19) provides a diagnostic fingerprint and exhibits a clear LO–TO splitting (673.5 vs. $806.6{\mathrm{cm}}^{-1}$). For *r*-BN, the most distinctive feature is the coincident strong Raman/IR peak of the high-frequency *E* mode at $1418.5{\mathrm{cm}}^{-1}$ (R ≈ 0.85; IR ≈ 0.97), which in the IR spectra shows a very large LO–TO separation (1418.5 vs. $1647.3{\mathrm{cm}}^{-1}$); a mid-frequency IR-active pair is also present at 695.8/$804.2{\mathrm{cm}}^{-1}$, while Raman activity in this range is negligible. Finally, *b*-BN displays the most complex pattern: in the high-frequency region, it shows more than one $E^{\prime }$ feature, including a strong TO at $1416.9{\mathrm{cm}}^{-1}$ (R ≈ 0.76, IR ≈ 0.86) and a weaker TO at $1428.1{\mathrm{cm}}^{-1}$ (R ≈ 0.12, IR ≈ 0.23), with LO counterparts at $1426.7{\mathrm{cm}}^{-1}$ and $1647.0{\mathrm{cm}}^{-1}$ (IR ≈ 0.98, R ≈ 0.88), evidencing a pronounced LO–TO splitting. In the mid-frequency range, an $A_{2}^{\prime \prime }$ mode appears with TO/LO at 697.9/$803.5{\mathrm{cm}}^{-1}$ (both IR-active, ≈ 0.14), while a second $A_{2}^{\prime \prime }$ pair near $793.1{\mathrm{cm}}^{-1}$ carries negligible intensity. These features are absent in the other polymorphs and provide clear diagnostics for *b*-BN. Overall, the combination of Raman spectra (Figure 3), the separate TO and LO panels of the IR spectra (Figure 4), and the LO–TO splittings listed in Table 4 provides a robust spectroscopic fingerprint set for distinguishing the four BN polymorphs, enabling direct matching between experimental Raman/IR data and computed fingerprints and offering a practical route for BN polytype identification.

### 3.5. Comparison with Experiment and Internal Consistency

The structural parameters obtained from both our PW/ONCV + D2 and PBE/PAW + D2 calculations are in good agreement with experimental data. Complete PW/ONCV + D2 structural data for all polymorphs are provided in the Supplementary Materials (Table S1). For *h*-BN, PW/ONCV + D2 gives a=2.486 Å and c=5.813 Å (Table S1), while PBE/PAW + D2 yields a=2.512 Å and c=6.179 Å (Table 1); both sets compare well with experimental averages of a=2.503 Å and c=6.661 Å [2,15]. Similarly, for *r*-BN we find a=2.485 Å, c=8.620 Å (PW/ONCV + D2) and a=2.511 Å, c=9.168 Å (PBE/PAW + D2), bracketing the experimental values a=2.504 Å, c=10.008 Å [2]. For *e*-BN and *b*-BN, no direct measurements exist, but our results are consistent with earlier theoretical studies [9,15]. In all cases, the in-plane lattice constant *a* is very well reproduced, while the interlayer parameter *c* is more sensitive to the treatment of dispersion, in line with earlier analyses [2].

Experimental optical band gaps of *h*-BN and *r*-BN are typically reported as E≈5.9–6.0 eV and E≈5.7 eV, respectively [3]. Our semilocal values are systematically smaller (Table 1), ranging from 3.9 to 4.6 eV depending on stacking and functional, which is expected given the well known underestimation of band gaps in GGA and LDA. Still, the relative ordering is reproduced: *r*-BN has a narrower gap than *h*-BN, with *e*-BN and *b*-BN lying in between. This trend is consistent with previous all-electron and diffusion Monte Carlo studies [4,9], indicating that the stacking dependence of the band gap is already well captured at the DFT level, even if absolute values require quasiparticle or excitonic corrections for quantitative agreement.

Turning to vibrational properties, the high-frequency TO Raman modes are well documented experimentally. For *h*-BN, the $E_{2g}$ line at ≈1360–$1366{\mathrm{cm}}^{-1}$ has long been established [7,17], while for *r*-BN, Raman and IR measurements show comparable high-frequency activity near 1365–$1370{\mathrm{cm}}^{-1}$ [2,3]. Our PW/ONCV + D2 results reproduce these signatures, yielding $1415.5{\mathrm{cm}}^{-1}$ for *h*-BN and $1418.5{\mathrm{cm}}^{-1}$ for *r*-BN. The corresponding LO partners are infrared-active: IR reflectivity and absorption studies confirm their presence and the associated LO–TO splitting [2,7,17], though the precise values vary with sample quality and measurement geometry. In particular, the well-known reststrahlen band of *h*-BN, widely exploited in nanophotonics, originates from the large LO–TO separation of the in-plane $E_{1u}$ phonons and provides strong indirect evidence for the large splitting predicted here [2]. In addition, Raman studies of epitaxial *h*-BN films have reported weaker spectral features near 1590–$1610{\mathrm{cm}}^{-1}$ [18], which are consistent with the high-frequency LO branches predicted in our calculations (e.g., $1642.9{\mathrm{cm}}^{-1}$ for *e*-BN, $1647.3{\mathrm{cm}}^{-1}$ for *r*-BN, and $1647.0{\mathrm{cm}}^{-1}$ for *b*-BN).

At mid frequencies, infrared data resolve the out-of-plane $A_{2u}$ branch of *h*-BN in the ≈670–$810{\mathrm{cm}}^{-1}$ window and IR-active $A_{1}$ features in *r*-BN around 700–$800{\mathrm{cm}}^{-1}$, both reproduced by our computed TO/LO pairs [2,3,17]. For *b*-BN and *e*-BN, experimental information remains scarce. Weak additional IR features in mixed-phase BN samples [1,8] have been tentatively linked to $A_{2}^{\prime \prime }$ activity in the 730–$800{\mathrm{cm}}^{-1}$ range, consistent with our predicted modes at 697.9/$803.5{\mathrm{cm}}^{-1}$ for *b*-BN. The sharp high-frequency $E^{\prime }$ mode of *e*-BN predicted at $1420.9{\mathrm{cm}}^{-1}$ has not yet been directly observed but is qualitatively compatible with occasional Raman signals from highly symmetric BN domains [19].

To quantify this correspondence, Table 5 summarizes the comparison between our computed Γ-point phonon frequencies and representative experimental benchmarks. For *h*-BN, the Raman $E_{2g}$ line differs by only +3.6%, while the infrared-active $A_{2u}$ TO and LO modes deviate by less than 1% from the experimental 670 and $810{\mathrm{cm}}^{-1}$ values, respectively [17]. For *r*-BN, the main Raman/IR feature near 1365–$1370{\mathrm{cm}}^{-1}$ agrees within 4% of our $1418.5{\mathrm{cm}}^{-1}$ result [2,3]. For *b*-BN (Bernal), direct Raman/IR benchmarks at Γ are not yet established. Instead, deep-UV cryomicroscopy combined with second-harmonic-generation (SHG) mapping identifies AB-stacked domains through a PL line at 6.035 eV, confirming their non-centrosymmetric nature [19]. This PL line arises from excitonic emission rather than the fundamental electronic gap but serves as a robust experimental fingerprint of the AB phase. Overall, the observed deviations remain below 5%, demonstrating excellent quantitative agreement between our calculations and experiment.

To further verify the robustness of our approach, Table 6 compares representative Γ-point modes obtained using the two computational setups, PBE/PAW + D2 and PW/ONCV + D2, each evaluated at its own fully relaxed lattice parameters and internal coordinates (i.e., method-consistent geometries). Both high-frequency in-plane (bond-stretching) and mid-frequency out-of-plane modes were analyzed. The frequency differences remain below ∼7%, the largest discrepancies corresponding to softer out-of-plane branches that are most sensitive to the exchange–correlation functional. These small, systematic shifts are well within the known spread between LDA↔GGA and NC↔PAW formalisms, demonstrating that the mixed PAW→ONCV workflow preserves the relative stack-dependent fingerprints while ensuring reproducible Raman/IR intensities.

Overall, both the structural and vibrational comparisons confirm that the computational framework reliably captures the stacking-dependent properties of $sp^{2}$-bonded BN. The excellent match with available experimental data and the consistent trends across methods validate the present approach as a transferable reference for the vibrational and optical signatures of all BN polytypes.

## 4. Summary

We presented a comprehensive first-principles framework for identifying layered BN polymorphs—*e*-BN (AA), *h*-BN (${\mathrm{AA}}^{\prime }$), *r*-BN (ABC), and *b*-BN (AB)—directly from Raman and infrared measurements. By computing symmetry-resolved phonons and mode activities at Γ with consistent settings (PW/ONCV + D2 for intensities; cross-checked with PBE/PAW + D2 for structures), we established a compact set of stack-specific fingerprints that can be read from the spectra. In practice: (i) *e*-BN is singled out by a single, sharp high-frequency $E^{\prime }$ line (TO near $1420.9{\mathrm{cm}}^{-1}$) with a large IR LO partner and little mid-frequency activity; (ii) *h*-BN shows the familiar Raman $E_{2g}$ peak (TO ≈ $1415.5{\mathrm{cm}}^{-1}$ in our calculations) and a distinctive out-of-plane $A_{2u}$ infrared mode with a clear TO/LO pair in the 670–$810{\mathrm{cm}}^{-1}$ range–an immediate mid-frequency fingerprint; (iii) *r*-BN exhibits a coincident high-frequency *E* feature that is strong in both Raman and IR (TO ≈ $1419{\mathrm{cm}}^{-1}$; its LO partner lies at $1647.3{\mathrm{cm}}^{-1}$), together with additional IR-active $A_{1}$ modes around 700–$800{\mathrm{cm}}^{-1}$; (iv) *b*-BN shows the most complex pattern: several high-frequency $E^{\prime }$ TO/LO pairs (1417–$1428{\mathrm{cm}}^{-1}$) of different strengths plus a robust mid-frequency $A_{2}^{\prime \prime }$ branch (TO near $698{\mathrm{cm}}^{-1}$ with an LO partner near $804{\mathrm{cm}}^{-1}$) that does not appear in the other stackings. We provide normalized intensities (per formula unit) and explicit TO/LO values to enable one-to-one comparison with measured spectra and to facilitate the interpretation of samples that contain more than one stacking. As practical rules of thumb: a strong mid-frequency IR line flags *h*-BN or *b*-BN; a single, intense high-frequency Raman line with weak mid-frequency response points to *e*-BN; and simultaneous strong Raman and IR activity at high frequency indicates *r*-BN. These decision rules, grounded in symmetry analysis and intensity trends, form a usable toolbox for BN polytype identification and are readily transferable to other layered $sp^{2}$ materials where stacking controls vibrational and optical responses.

## Figures and Tables

**Table 1:** Comparison of structural parameters, cohesive energy ($E_{c}$), and electronic band gap (*E*) for layered BN polymorphs with and without vdW corrections. Where possible, experimental and/or theoretical values from the literature are provided for comparison.

Method	*a* [Å]	*c* [Å]	$E_{c}$ [eV]	*E* [eV]
**e-BN (AA)**				
without vdW	2.514	5.059	7.064	4.12 (indirect, K–Γ)
with vdW	2.511	3.383	7.181	4.63 (indirect, K–Γ)
lit. theory	2.476 [2]	3.476 [2]	–	–
**h-BN (${\mathrm{AA}}^{\prime }$)**				
without vdW	2.515	9.136	7.065	4.25 (indirect, K–Γ)
with vdW	2.512	6.179	7.205	4.10 (indirect, K–M)
lit. theory	2.478 [2]	6.354 [2]	7.055 [15]	4.25 [3]
lit. exp.	2.503 [2]	6.661 [2]	–	5.97 [3]
**r-BN (ABC)**				
without vdW	2.515	13.743	7.065	4.24 (indirect, K–Γ)
with vdW	2.511	9.168	7.207	3.94 (indirect, K–M)
lit. theory	2.476 [2]	9.679 [2]	–	4.21 [3]
lit. exp.	2.504 [2]	10.008 [2]	–	5.7 [3]
**b-BN (AB)**				
without vdW	2.514	9.229	7.065	4.11 (indirect, K–Γ)
with vdW	2.511	6.117	7.207	3.98 (direct, K–K)
lit. theory	2.477 [2]	6.319 [2]	–	–

**Table 2:** Phonon frequencies (in ${\mathrm{cm}}^{-1}$) at the Γ point for each BN polymorph, calculated using DFPT with and without vdW corrections. Each entry lists the frequency shift from no-vdW to vdW, followed by the irreducible representation and mode activity: infrared (I), Raman (R), both (I + R), or silent (S). Only optical phonons are included, with vdW-corrected values shown second in each pair.

*e*-BN		*h*-BN		*r*-BN		*b*-BN	
Freq.(no → vdW)	Mode	Freq. (no → vdW)	Mode	Freq. (no → vdW)	Mode	Freq. (no → vdW)	Mode
783 → 757.2	$A_{2}^{\prime \prime }$ (I)	≈0 → 39.6	$E_{2g}$ (R)	752.5 → 717.4	$A_{1}$ (I + R)	≈0→ 48.5	$E^{\prime }$ (I + R)
1343.5 → 1352.5	$E^{\prime }$ (I + R)	52.9 → 182.8	$B_{1g}$ (S)	1384.5 → 1395.8	*E* (I + R)	55.2 → 180.0	$A_{2}^{\prime \prime }$ (I)
		781.1 → 723.0	$A_{2u}$ (I)			781.6 → 732.0	$A_{2}^{\prime \prime }$ (I)
		803.0 → 792.1	$B_{1g}$ (S)			803.0 → 793.9	$A_{2}^{\prime \prime }$ (I)
		1341.6 → 1349.3	$E_{1u}$ (I)			1343.6 → 1351.9	$E^{\prime }$ (I + R)
		1341.6 → 1350.3	$E_{2g}$ (R)			1343.6 → 1358.7	$E^{\prime }$ (I + R)

**Table 3:** Optical phonon frequencies at the Γ point for each BN polymorph, including vdW corrections. Frequencies are in ${\mathrm{cm}}^{-1}$. Each mode is labeled with its irreducible representation and activity type: infrared (I), Raman (R), both (I + R), or silent (S). All values are from the PW/ONCV calculations; for *r*-BN a primitive rhombohedral cell is used. No LO–TO splitting is reported here (TO values shown).

*e*-BN		*h*-BN		*r*-BN		*b*-BN	
Freq.	Mode	Freq.	Mode	Freq.	Mode	Freq.	Mode
737.8	$A_{2}^{\prime \prime }$ (I)	86.7	$E_{2g}$ (R)	695.8	$A_{1}$ (I + R)	92.4	$E^{\prime }$ (I + R)
1420.9	$E^{\prime }$ (I + R)	242.6	$B_{1g}$ (S)	1418.5	*E* (I + R)	247.2	$A_{2}^{\prime \prime }$ (I)
		673.5	$A_{2u}$ (I)			697.9	$A_{2}^{\prime \prime }$ (I)
		789.0	$B_{1g}$ (S)			793.1	$A_{2}^{\prime \prime }$ (I)
		1412.5	$E_{1u}$ (I)			1416.9	$E^{\prime }$ (I + R)
		1415.5	$E_{2g}$ (R)			1428.1	$E^{\prime }$ (I + R)

**Table 4:** Calculated Raman and infrared (IR) intensities for each BN polymorph at the Γ point, normalized separately for IR and Raman to the largest value among all four polymorphs. For *h*-BN and *b*-BN, raw intensities were first divided by two (four atoms per cell) before normalization. Frequencies are in ${\mathrm{cm}}^{-1}$ and normalized intensities are dimensionless. All values are obtained from the PW/ONCV + D2 calculations; for *r*-BN a primitive rhombohedral cell is used, with LO–TO splitting shown explicitly for IR-active modes.

*e*-BN			*h*-BN			*r*-BN			*b*-BN		
Freq.	IR	Raman	Freq.	IR	Raman	Freq.	IR	Raman	Freq.	IR	Raman
737.8 (TO)	0.07		86.7		0.00	695.8 (TO)	0.15	0.06	92.4 (TO)	0.00	0.00
797.2 (LO)	0.07		242.6			804.2 (LO)	0.15	0.06	92.6 (LO)	0.00	0.00
1420.9 (TO)	0.98	1.00	673.5 (TO)	0.19		1418.5 (TO)	0.97	0.85	247.2 (TO)	0.00	
1642.9 (LO)	0.98	1.00	806.6 (LO)	0.19		1647.3 (LO)	0.97	0.84	249.3 (LO)	0.00	
			788.5						697.9 (TO)	0.14	
			1412.5 (TO)	1.00					803.5 (LO)	0.14	
			1650.5 (LO)	1.00					793.1 (TO)	0.00	
			1415.5		0.83				793.1 (LO)	0.00	
									1416.9 (TO)	0.86	0.76
									1426.7 (LO)	0.00	0.00
									1428.1 (TO)	0.23	0.12
									1647.0 (LO)	0.98	0.88

**Table 5:** Γ-point optical phonon frequencies (${\mathrm{cm}}^{-1}$) at ∼300 K for $sp^{2}$-bonded BN polytypes: experiment vs. this work (PW/ONCV + D2). Deviations are defined as $\left(\omega_{\mathrm{calc}}-\omega_{\exp}\right)/\omega_{\exp}\times 100\%$. For *h*-BN, we report separate TO and LO values for the IR-active $A_{2u}$ branch. For *b*-BN, no consensus Raman/IR phonon frequencies at Γ are available to date; we therefore list the deep-UV PL line at 6.035 eV as an AB-stacking diagnostic.

Polytype	Mode (Sym.)	Experiment	This Work	Δ (${\mathrm{cm}}^{-1}$)	Δ (%)	Source
*h*-BN	$E_{2g}$ (R)	1366	1415.5	+49.5	+3.6	[17]
*h*-BN	$A_{2u}$ (IR, TO)	670	673.5	+3.5	+0.5	[17]
*h*-BN	$A_{2u}$ (IR, LO)	810	806.6	−3.4	−0.4	[17]
*r*-BN	*E* (R/IR)	1365–1370	1418.5	+48.5–53.5	+3.5–3.9	[2,3]
*b*-BN	(diagnostic, PL)	6.035 eV	–	–	–	[19]

**Table 6:** Internal consistency cross-check of selected Γ-point phonons (${\mathrm{cm}}^{-1}$) obtained with PBE/PAW + D2 and PW/ONCV + D2, each at its own fully relaxed geometry. Here, $\Delta =\omega_{\mathrm{ONCV}}-\omega_{\mathrm{PAW}}$. IR-active modes are reported as TO values for consistency.

Polytype	Mode	$\omega_{\mathrm{PAW}}$	$\omega_{\mathrm{ONCV}}$	Δ (${\mathrm{cm}}^{-1}$)	Δ (%)
**High-frequency in-plane modes**					
*e*-BN	$E^{\prime }$ (I + R)	1352.5	1420.9	+68.4	+5.1
*h*-BN	$E_{2g}$ (R)	1350.3	1415.5	+65.2	+4.8
*r*-BN	*E* (I + R)	1395.8	1418.5	+22.7	+1.6
*b*-BN	$E^{\prime }$ (I + R)	1358.7	1416.9	+58.2	+4.3
**Mid-frequency out-of-plane modes**					
*e*-BN	$A_{2}^{\prime \prime }$ (I)	757.2	737.8	−19.4	−2.6
*h*-BN	$A_{2u}$ (I)	723.0	673.5	−49.5	−6.8
*r*-BN	$A_{1}$ (I + R)	717.4	695.8	−21.6	−3.0
*b*-BN	$A_{2}^{\prime \prime }$ (I)	732.0	697.9	−34.1	−4.7

## Data Availability

The original contributions presented in this study are included in the article. Further inquiries can be directed to the corresponding author.

## Supplementary Materials

The following supporting information can be downloaded at: https://www.mdpi.com/article/10.3390/nano15201567/s1, Table S1: Relaxed lattice parameters and cohesive energies $E_{c}$ (per atom) of BN polymorphs obtained with PW/ONCVP + D2. Relative stabilities, ΔE, are given with respect to the most stable phase, *r*-BN.
