# Supplementary material for: Raman and Infrared Signatures of Layered Boron Nitride Polytypes: A First-Principles Study
Source: Nanomaterials (Basel). 2025 Oct 15;15(20):1567. doi: 10.3390/nano15201567 (PMC12566195; doi:10.3390/nano15201567)
Supplement: Supplementary file 1 [file nanomaterials-15-01567-s001.zip › nanomaterials-3872269-supplementary.pdf]

Supplementary Materials for: Raman and Infrared Signatures of Layered Boron Nitride Polytypes: A First-Principles Study

Priyanka Mishra and Nevill Gonzalez Szwacki
Faculty of Physics, University of Warsaw, Pasteura 5, PL-02093 Warsaw, Poland

Table S1 reports PW/ONCVP + D2 relaxed lattice parameters (a, c) and cohesive energies per atom for the four BN polymorphs (e-, h-, r-, and b-BN). Relative stabilities ΔE are referenced to the most stable phase (r-BN).

TABLE S1. Relaxed lattice parameters and cohesive energies E_c (per atom) of BN polymorphs obtained with PW/ONCVP + D2. Relative stabilities, ΔE, are given with respect to the most stable phase, r-BN.

Phase	a [Å]	c [Å]	E_c [eV/atom]	ΔE [eV/atom]
e-BN (AA)	2.483	3.153	8.135	0.048
h-BN (AA′)	2.486	5.813	8.178	0.005
r-BN (ABC)	2.485	8.620	8.183	0.000
b-BN (AB)	2.485	5.753	8.183	0.000
